# ﻿Descriptions of four species of Polyxenida Verhoeff, 1934 (Diplopoda, Penicillata) from China, including one new species and one new record

**DOI:** 10.3897/zookeys.1223.135808

**Published:** 2025-01-06

**Authors:** Yadong Wang, Ai Jin, Shichen Gao, Jiajia Wang, Yan Dong

**Affiliations:** 1 College of Biology and Food Engineering, Chuzhou University, Chuzhou 239000, China Chuzhou University Chuzhou China; 2 School of Life Sciences, Anhui University, Hefei 230601, China Anhui University Hefei China

**Keywords:** China, COI, *Eudigraphis*, identification key, *Lophoturus*, molecular phylogeny, millipede, *Polyxenus*, taxonomy

## Abstract

This study describes a new species of Polyxenida from China, *Lophoturussineprocessus***sp. nov.**, along with a species newly recorded from China: *Eudigraphisnigricans* (Miyosi, 1947), and provides additional descriptions of *Eudigraphissinensis* Ishii & Liang, 1990 and *Polyxenushangzhoensis* Ishii & Liang, 1990. The study conducted mitochondrial cytochrome *c* oxidase subunit I (COI) sequencing for all four species and constructed a phylogenetic tree based on the molecular data. The comprehensive morphological descriptions and molecular analyses confirm the addition of one new species and one newly recorded species for the Polyxenida fauna of China, elevating the total number of known Polyxenida species in the country from 10 to 12. The study also includes an identification key for Polyxenida species in China.

## ﻿Introduction

The order Polyxenida Verhoeff, 1934 belongs to the class Diplopoda, subclass Penicillata, and is the sole extant order within the subclass (Short and Vahtera 2017). Polyxenida possess elongated bodies typically ranging from 1.2 to 6 millimeters, with a soft, non-calcified cuticle (Enghoff et al. 2015). Members of this group inhabit diverse environments, with most species found under bark, in moist leaf litter, and decaying wood. Some specialized species reside in coastal environments (Huynh and Veenstra 2018a) or caves (Nguyen Duy-Jacquemin 2014). Polyxenida primarily feed on humus, algal films, lichens, and fungal hyphae (Vohland and Hamer 2013; Karasawa et al. 2020), and are preyed upon by predators such as spiders, ants, and centipedes. Currently, there are 3 families, 32 genera, and approximately 190 species of Polyxenida known worldwide, constituting approximately 1.5% of all known millipede species.

Research on the order Polyxenida in China began relatively late. In 1990, Japanese scholar Ishii first reported *Eudigraphistaiwaniensis* Ishii, 1990 and *Lophoturusokinawai* (Nguyen Duy-Jacquemin & Condé, 1982), which are distributed in southern Taiwan (Ishii 1990). In the same year, Ishii collaborated with Liang to describe two new species from Hangzhou, Zhejiang: *Polyxenushangzhoensis* Ishii & Liang, 1990, and *Eudigraphissinensis* Ishii & Liang, 1990 (Ishii and Liang 1990). In 2000, Ishii and Yin reported six new species and one yet unnamed species from the northwest of Yunnan, Southwest China: *Lophoturusjianshuiensis* Ishii & Yin, 2000, *Monographisyunnanensis* Ishii & Yin, 2000, *Monographisbaihualingensis* Ishii & Yin, 2000, *Eudigraphisxishuangbanna* Ishii & Yin, 2000, *Polyxenusanophthalius* Ishii & Yin, 2000, *Polyxenustriocellatus* Ishii & Yin, 2000, and *Polyxenus* sp. (Ishii and Yin 2000). Since then, there have been no further records of new Polyxenida from China.

China boasts a diverse array of climates and terrains, along with abundant natural vegetation, supporting a wide variety of animal species (Yang and Bu 2021). However, only 10 species of Polyxenida, belonging to 2 families and 4 genera, have been reported from China. Consequently, the understanding of Polyxenida species diversity in China remains limited, significantly lagging behind other arthropod groups.

**Figure 1.**
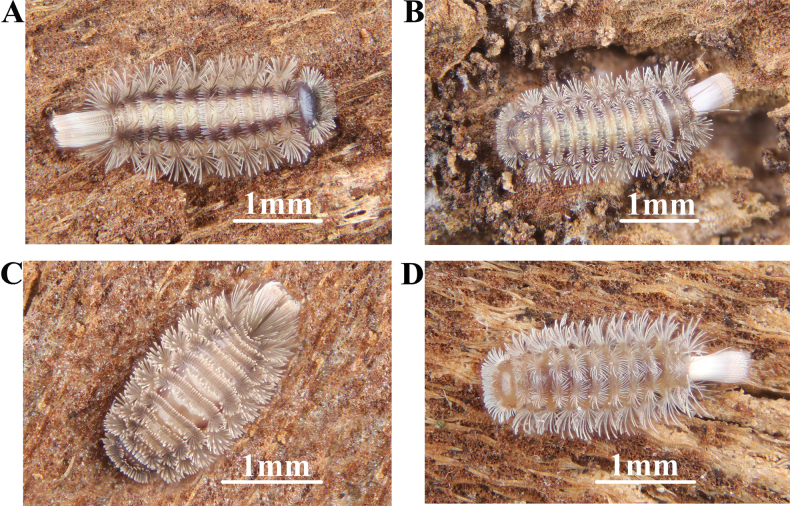
**A***Eudigraphisnigricans* (Miyosi, 1947) **B***Eudigraphissinensis* Ishii & Liang, 1990 **C***Polyxenushangzhoensis* Ishii & Liang, 1990 **D***Lophoturussineprocessus* sp. nov. All Polyxenida photographed by Y. D. Wang.

In this study, the authors collected four Polyxenida species from four regions in China: *Eudigraphisnigricans* (Miyosi, 1947), *Eudigraphissinensis* Ishii & Liang, 1990, *Polyxenushangzhoensis* Ishii & Liang, 1990, and *Lophoturussineprocessus* sp. nov. (Fig. 1), including one new species and one new record for China. The authors provided detailed morphological descriptions for these four species. Furthermore, by using DNA sequence data, we constructed a molecular phylogenetic tree to supplement the morphological analysis and compiled a key to the species of Polyxenida from China. This study represents the first report of a new species and a newly recorded species in China since Ishii’s 2000 publication, increasing the known Polyxenida species in China from ten to twelve.

## ﻿Material and methods

### ﻿Specimen collection and morphological observations

Y. D. Wang collected the Polyxenida specimens and preserved them in 75% ethanol. Table 1 presents the specimen collection time and location information. The authors selected specimens for measurement, clearing, and mounting on slides using Hoyer’s medium. They examined the mounted specimens using a ZEISS Axioscope 5 microscope (Germany). The Animal Collection of Chuzhou University will receive all specimens used for morphological observation, except for those severely damaged during dissection. Table 1 lists the corresponding voucher numbers.

**Table 1.** Specimen information including morphological identification, voucher number, collection details, and GenBank accession numbers.

Species	Voucher	Sampling sites	Sampling time	Accession No.
*Eudigraphisnigricans*	CZHZS3	Su Causeway, West Lake, Hangzhou, Zhejiang Province, China	April 23, 2024	PQ141065
*Eudigraphissinensis*	CZHZS1	Su Causeway, West Lake, Hangzhou, Zhejiang Province, China	April 23, 2024	PQ142931
	CZNJS1	Zhongshan Mountain National Park, Nanjing City, Jiangsu Province, China	April 12, 2024	PQ142932
*Polyxenushangzhoensis*	CZCZS1	Langya Mountain, Chuzhou City, Anhui Province, China	March 12, 2024	PQ142930
*Lophoturussineprocessus*	CZYNS1	Menglai Rainforest Health Theme Park, Jinghong City, Xishuangbanna Dai Autonomous Prefecture, Yunnan Province, China	August 20, 2023	PQ142933

The naming of leg segments follows the convention established by Manton (1956).

### ﻿DNA extraction and polymerase chain reaction (PCR) amplification

Through meticulous morphological examination, a total of five individuals (corresponding to five voucher numbers) were selected for DNA extraction. Total genomic DNA was extracted from each specimen using a QIAamp® DNA Micro Kit (Qiagen, Germany), following the manufacturer’s protocol. A partial region of the mitochondrial cytochrome *c* oxidase subunit I (COI) was amplified by PCR using the following primers: CO1CF and CO1CR (Wang et al. 2022). PCRs were carried out in 12.5 μl Taq PCR Master Mix (2X, Blue Dye) (Sangon Biotech, Shanghai), 0.5 μl of each primer pair, 9.5 μl distilled water, and 2 μl sample DNA. The PCR cycle program included an initial denaturation at 94 °C for 3 min, 34 cycles of denaturation at 94 °C for 30 sec, annealing at 50 °C for 30 sec, elongation at 72 °C for 1 min, and a final extension at 72 °C for 10 min, with storage at 4 °C. The PCR products were directly sent to General Biology (Anhui) for purification and sequencing. New sequences were deposited in GenBank. The accession numbers are listed in Table 1.

### ﻿Phylogenetic analysis

In the phylogenetic analyses, we selected all COI sequences from the ingroup genera *Polyxenus* (*P.argentifer*, MN073978; *P.fasciculatus*, MN073933; *P.lagurus*, MN073968; *P.* sp., MN073971), *Lophoturus* (*L.boondallus*, MG204536; *L.queenslandicus*, MG204535; *L.* sp., MT679994), and *Eudigraphis* (*E.nigricans*, LC010896; *E.* sp., LC010908) available on the National Center for Biotechnology Information (NCBI); each sequence was at least 600 base pairs (bp) in length. Two species from closely related families, *Glomerisbalcanica* (PP475128) and *Rhopalomerissauda* (MT749404), were used as outgroups.

Sequence alignment was performed using MAFFT 7 (Katoh and Standley 2013), resulting in a COI alignment containing no gaps. Unrooted phylogenetic trees were constructed using maximum likelihood (ML) and Bayesian inference (BI) methods. The ML analysis was conducted using IQ-TREE 2 software (Minh et al. 2020), employing the General time reversible (GTR) model for COI sequence evolution. Bootstrap proportions (BP) for the ML analysis were assessed using 1000 replicates. Bayesian analyses were performed in MrBayes v. 3.2.7, implemented in the CIPRES Science Gateway (Miller et al. 2010https://www.phylo.org). The BI analysis utilized the HKY85+I+G model and consisted of running four simultaneous chains for 100,000 generations, with tree sampling every 1000 generations and a 25% burn‐in. Convergence of the runs was verified by an average standard deviation of <0.01.

## ﻿Taxonomy

### ﻿Class Diplopoda de Blainville in Gervais, 1844

**Subclass Penicillata Latreille, 1831**

**Order Polyxenida Verhoeff, 1934**

**Family Polyxenidae Lucas, 1840**

**Subfamily Monographinae Condé, 2008**

#### ﻿Genus Eudigraphis

Taxon classificationAnimaliaPolyxenidaPolyxenidae

Silvestri, 1948

C2598706-7F56-5C1E-8502-EBB6260461EC

##### Type species.

*Eudigraphisjaponica* Silvestri, 1948.

##### Genus diagnosis.

Adults with 13 pairs of legs and 8 ommatidia on each side. Dorso-medial (ornamental) barbate trichomes in one or two rows arranged transversely dorsal and anterior to the penicil of the telson. A small setiform hair with a round base is present on Tarsus II. *Eudigraphis* can be recognized by the presence of tergal trichomes in two lateral clusters plus an uninterrupted single posterior transverse row (Silvestri 1948).

##### Included species.

*Eudigraphistakakuwai* (Miyosi, 1947), *E.nigricans* (Miyosi, 1947), *E.kinutensis* (Haga, 1950), *E.sinensis* Ishii & Liang, 1990, *E.taiwaniensis* Ishii, 1990, *E.xishuangbanna* Ishii & Yin, 2000.

#### ﻿ Eudigraphis nigricans

Taxon classificationAnimaliaPolyxenidaPolyxenidae

(Miyosi, 1947)

25AC9402-D4FC-554E-85AF-87CCFB03AA27

Fig. 2

Monographis takakuwai nigricans Miyoshi, 1947: 7; Takashima and Haga 1950: 23, fig. 2.
Eudigraphis takakuwai nigricans : Ishii 1988: 957, figs 10, 11; Nguyen Duy-Jacquemin and Geoffroy 2003: 99; Mimizu Club and Minagoshi 2013: 44, 3 photos; Kawano 2017: 14, figs 1–12.
Eudigraphis nigricans : Ishii and Tamura (1995) described this species on page 233; Ishii (1999) further discussed it on page 212; Ishii (2002) provided additional information on page 289; Karasawa et al. (2020) recently reviewed the species on page 94 and presented an illustration in figure 4D.

##### Material examined.

China • 4♂2♀; Zhejiang, Hangzhou, West Lake; 30°23'58"N, 120°14'04"E; 23 April 2024; Y. D. Wang leg.; GenBank: PQ141065; CBF CZHZS3.

##### Diagnosis.

Ground color of body pale yellowish-brown in dorsal view, but head black. Body dorsally with a pair of belt-like dark brown markings that run slightly off from both of the rims of each segment of body. Antennal article VI has 3 thick basiconic sensilla, and article VII has 2 thick basiconic sensilla.

##### Description.

**Female.** With 13 pairs of legs. Measurements: Body length 3.4 mm, caudal bundle 0.5 mm.

**Figure 2.**
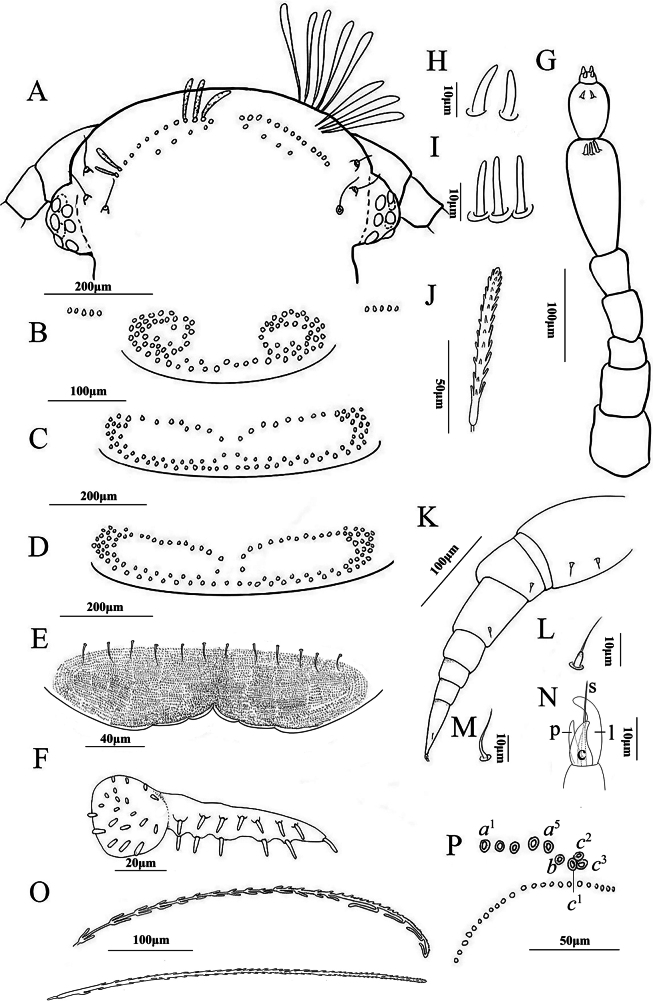
*Eudigraphisnigricans* (Miyosi, 1947) adult female **A** head **B** collum **C** and **D** tergites showcasing the pattern of trichome insertions **C** tergite II **D** tergite III **E** clypeo-labrum **F** gnathochilarium **G** antenna **H** sensilla on articles VII **I** sensilla on articles VI **J** anterior vertex trichome **K** left 13^th^ leg **L** typical setae of coxa, prefemur, and femur **M** small setiform hair on tarsus II **N** telotarsus structure with processes indicated: c: claw, l: lamella, p: posterior process, s: setiform process **O** hooked caudal trichome **P** pattern of insertions of dorso-medial trichomes on telson. Scale bars: 200 μm (**A, C, D**); 100 μm (**B, G, K, O**); 50 μm (**J, P**); 40 μm (**E**); 20 μm (**F**); 10 μm (**H, I, L, M, N**).

***Head*** (Fig. 2A): Eyes comprising 8 ommatidia. The posterior vertex possesses one pair of tufts each arranged in two rows; each anterior row consists of 13 trichomes, and the posterior row consists of 4 (Fig. 2A). Trichomes are depicted in Fig. 2J. Trichobothria are equal in size and arranged in an isosceles triangle formation (Fig. 2A). The gnathochilarium’s lateral palps are twice the length of the medial palp. Lateral palps with 13 sensilla, medial palp with 20 sensilla (Fig. 2F). The labrum’s anterior margin is granulated and armed with 3+3 lamellar teeth, and the clypeo-labrum with 6+6 setae (Fig. 2E).

***Antennae***: Long antennae with proportions of antennal articles as depicted in Fig. 2G. Antennal article VIII with 4 sensory cones, while article VI with 3 thick basiconic sensilla (Fig. 2I); article VII with 2 thick basiconic sensilla (Fig. 2H).

***Trunk***: Collum with one pair of tufts, each consisting of 44 trichomes, lateral protuberance of collum with 5 trichomes in a row (Fig. 2B). Tergite II, with one pair of tufts each consisting of 45 trichomes (Fig. 2C) connected by a continuous posterior row of trichomes. Tergite III, with one pair of tufts each composed of 52 trichomes (Fig. 2D) and connected by a continuous posterior row of trichomes. Tergites II–X have the same pattern of trichome insertions.

***Legs*** (Fig. 2K): Trochanter, post-femur, tibia, and tarsus I lack setae. Prefemur and femur each with 1 seta (Fig. 2L), coxa I with 1 seta, coxae II–XII with 3–4 setae, coxa XIII with 2 setae, small setiform hair on tarsus II shorter than telotarsus (Fig. 2M). Telotarus is composed of a posterior process, almost as long as the claw, lamella process and a setiform process are present (Fig. 2N).

***Telson***: Dorso-medial trichomes on each side consist of 5 sockets of trichome *a*^1–5^, a single trichome *b*, and three large protruding base sockets of trichome *c*^1–3^ (Fig. 2P). Beneath these, there are two bundles of caudal trichomes separated by a very narrow gap. The telson trichomes exist in two forms: those with hooks and those without hooks. The hooked trichomes most commonly with 2–4 hooks (Fig. 2O).

**Male.** With 13 pairs of legs. Measurements: Body length 3.2 mm, caudal bundle 0.47 mm. Lateral palps with 13 sensilla, medial palp with 21 sensilla. The anterior margin of the labrum is granulated; the clypeo-labrum with 6+6 setae. The collum features one pair of tufts consisting of 41 trichomes each. Tergites II and III with one pair of tufts comprised of 42 or 50 trichomes. Coxa I with 1 seta, coxae II–X with 3–4 setae, coxae XI–XII with 2 setae, and coxa XIII with 1 seta. The dorso-medial trichomes on each side are composed of trichomes *a*^1–4^, *b*, and *c*^1–3^.

##### Distribution.

China (Zhejiang), Japan.

##### Remarks.

This species closely resembles *Eudigraphistakakuwai* Miyosi, 1947, but differs in possessing a black head.

#### ﻿ Eudigraphis sinensis

Taxon classificationAnimaliaPolyxenidaPolyxenidae

Ishii & Liang, 1990

BC1A1BB6-6BF6-54D3-9509-FAC733832D70

Fig. 3

##### Material examined.

China • 3♂5♀; Zhejiang, Hangzhou, West Lake; 30°24'60"N, 120°13'72"E; 23 April 2024; Y. D. Wang leg.; GenBank: PQ142931; CBF CZHZS1; • 4♂3♀; Jiangsu, Nanjing, Zhongshan Mountain National Park; 32°08'47"N, 118°84'38"E; 12 April 2024; leg. Y. D. Wang leg.; GenBank: PQ142932; CBF CZNJS1.

##### Diagnosis.

Anterior margin of the labrum is not granulated and is equipped with 3+3 lamellar teeth on the anterior margin. Oval bases of setae on coxa, prefemur, and femur of each leg with some long spines at apex. Antennal article VI with 3 thick basiconic sensilla, 1 setiform sensillum, and 1 conical sensillum; article VII with 2 thick basiconic sensilla, 1 setiform sensillum, and 1 conical sensillum.

##### Description.

**Female.** With 13 pairs of legs. Measurements: Body length 2.8 mm, caudal bundle 0.45 mm.

**Figure 3.**
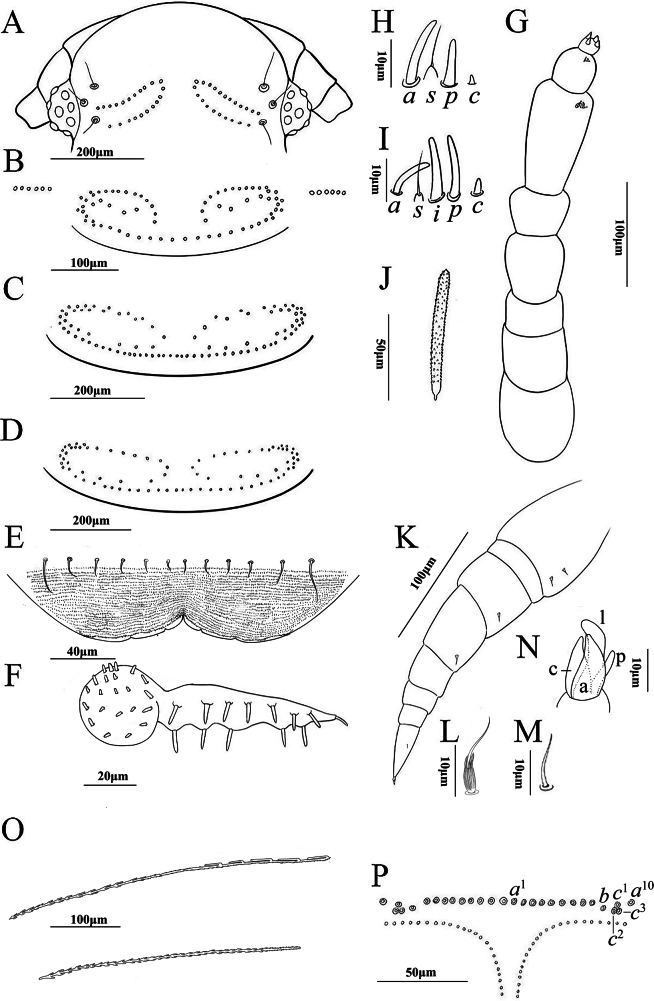
*Eudigraphissinensis* Ishii & Liang, 1990, adult female **A** head **B** collum **C** and **D** tergites showcasing the pattern of trichome insertions **C** tergite II **D** tergite III **E** clypeo-labrum **F** gnathochilarium **G** antenna **H** sensilla on articles VII **I** sensilla on articles VI **J** anterior vertex trichome **K** left 13^th^ leg **L** typical setae of coxa, prefemur, and femur **M** small setiform hair on tarsus II **N** telotarsus structure with processes indicated: a: anterior process, c: claw, l: lamella, p: posterior process **O** hooked caudal trichome **P** pattern of insertions of dorso-medial trichomes on telson. Scale bars: 200 μm (**A, C, D**); 100 μm (**B, G, K, O**); 50 μm (**J, P**); 40 μm (**E**); 20 μm (**F**); 10 μm (**H, I, L, M, N**).

***Head*** (Fig. 3A): Eyes comprising 8 ommatidia. The posterior vertex possesses one pair of tufts each arranged in two rows: each anterior row comprises 12 trichomes, while the posterior row contains 10 (Fig. 3A). The trichomes are depicted in Fig. 3J. The trichobothria are equal in size and arranged in an isosceles triangle configuration (Fig. 3A). The gnathochilarium’s lateral palps are twice the length of the medial palp. Lateral palps with 13 sensilla, medial palp with 22 sensilla (Fig. 3F). The anterior margin of the labrum is not granulated and is equipped with 3+3 lamellar teeth on the anterior margin. The clypeo-labrum with 6+6 setae (Fig. 3E).

***Antennae***: Long antennae with proportions of antennal articles as depicted in Fig. 3G. Antennal article VIII with 4 sensory cones, antennal article VI with 3 thick basiconic sensilla (*a*, *i*, and *p*), 1 setiform sensillum (*s*) situated between *a* and *i*, and 1 conical sensillum (*c*) behind *p* (Fig. 3I); article VII with 2 thick basiconic sensilla of *a* and *p*, 1 setiform sensillum (*s*) located between *a* and *p*, and 1 conical sensillum (*c*) behind *p* (Fig. 3H).

***Trunk***: Collum with one pair of tufts, each consisting of 35 trichomes, lateral protuberance of collum with 6 trichomes in a row (Fig. 3B). Tergites II, with one pair of tufts each consisting of 45 trichomes (Fig. 3C) connected by a continuous posterior row of trichomes. Tergites III, with one pair of tufts each composed of 40 trichomes (Fig. 3D) and connected by a continuous posterior row of trichomes. Tergites II–X exhibit a consistent pattern of trichome insertions.

***Legs*** (Fig. 3K): Trochanter, post-femur, tibia, and tarsus I lack setae. Prefemur and femur each with 1 seta, coxa I with 1 seta, coxa II with 2 or 3 setae, coxae III–XII with 3 setae, coxa XIII with 1 seta (Fig. 3L), oval bases of setae on coxa, prefemur, and femur of each leg with some long spines at apex. Small setiform hair on tarsus II shorter than telotarsus (Fig. 3M). Telotarsus is composed of an anterior process, with an enlarged base, almost as long as the claw. Lamella process and a posterior process are present (Fig. 3N).

***Telson***: Dorso-medial trichomes on each side consist of 10 sockets of trichome *a*^1–10^, a single trichome *b*, and three large protruding base sockets of trichome *c*^1–3^ (Fig. 3P). Two bundles of caudal trichomes are located beneath with a very narrow gap. The telson trichomes are of two types: those with hooks and those without hooks. The hooked trichomes of the caudal bundles most commonly possess 1–5 hooks (Fig. 3O).

**Male.** With 13 pairs of legs. Measurements: Body length 2.5 mm, caudal bundle 0.4 mm. Lateral palps with 13 sensilla, medial palp with 21 sensilla. The anterior margin of the labrum is not granulated and is armed with 3+3 lamellar teeth on the anterior margin. Clypeo-labrum with 6+6 setae. Collum each with one pair of tufts consisting of 32 trichomes; tergites II and III with one pair of tufts comprised of 42 or 39 trichomes. Coxa I with 1 seta, coxa II with 2 or 3 setae, coxae III–X with 3 setae, coxae XI–XII with 2 setae, coxa XIII with 1 seta.

##### Distribution.

China (Zhejiang, Jiangsu).

##### Remarks.

This species closely resembles *Eudigraphiskinutensis* Haga, 1950, but can be easily distinguished from the latter by the presence of 3+3 lamellar teeth on the anterior margin of the labrum (2+2 in *E.kinutensis*).

### ﻿Subfamily Polyxeninae Lucas, 1840

#### ﻿Genus Polyxenus

Taxon classificationAnimaliaPolyxenidaPolyxenidae

Latreille, 1802

DEF6B9EE-604D-5383-A53B-F775B81F3B7C

##### Type species.

*Polyxenuslagurus* Linnaeus, 1758.

##### Genus diagnosis.

Adults with 13 pairs of legs, ommatidia are typically present, although they may be absent in certain species. A fan of barbate trichomes is situated dorso-medially, anterior to the penicil. The two bundles of trichomes that form the caudal penicil are widely separated. *Polyxenus* can be recognized by the presence of two rows of trichomes on each tergite.

##### Included species.

*Polyxenusalbus* Pocock, 1894, *P.anacapensis* Pierce, 1940, *P.anophthalius* Ishii & Yin, 2000, *P.caudatus* Menge, 1854, *P.chalcidicus* Condé & Nguyen Duy-Jacquemin, 1971, *P.chilensis* Silvestri, 1903, *P.colurus* Menge, 1854, *P.conformis* Koch & Berendt, 1854, *P.fasciculatus* Say, 1821, *P.hangzhoensis* Ishii & Liang, 1990, *P.hawaiiensis* Silvestri, 1904, *P.koreanus* Ishii & Choi, 1988, *P.lagurus* (Linnaeus, 1758), *P.lapidicola* Silvestri, 1903, *P.lankaranensis* Short, 2020, *P.lepagei* Mello-Leitão, 1925, *P.lophurus* Menge, 1854, *P.macedonicus* Verhoeff, 1952, *P.miocenica* Srivastava, 2006, *P.oromii* Nguyen Duy-Jacquemin, 1996, *P.ovalis* Koch & Berendt, 1854, *P.paraguayensis* Silvestri, 1903, *P.platensis* Silvestri, 1903, *P.pugetensis* Kincaid, 1898, *P.rossi* Chamberlin, 1957, *P.senex* Mello-Leitão, 1925, *P.shinoharai* Ishii, 1983, *P.superbus* Silvestri, 1903, *P.triocellatus* Ishii & Yin, 2000, *P.tuberculatus* Pierce, 1940.

#### ﻿ Polyxenus hangzhoensis

Taxon classificationAnimaliaPolyxenidaPolyxenidae

Ishii & Liang, 1990

E5A4E1BC-7035-53B2-9916-C0DEE0B2F0B2

Fig. 4

##### Material examined.

China • 3♂4♀; Anhui, Chuzhou, Langya Mountain; 32°27'67"N, 118°30'01"E; 12 Mar. 2024; Y. D. Wang leg.; GenBank: PQ142930; CBF CZCZS1.

##### Diagnosis.

Five ommatidia in each eye. Number of trichomes: posterior vertex, 47–48; collum, 54–55; collum’s lateral protuberance, 4–5; tergite II, 56–62; tergite III, 60–64. Four dorso-medial trichomes on the head, posterior vertex comprising 2 complete rows of trichomes with no medial gap. Antennal article VI with 2 thick basiconic sensilla, 1 setiform sensillum, and 6 thin basiconic sensilla. Antennal article VII with 2 thick basiconic sensilla, 1 setiform sensillum, 4 thin basiconic sensilla, and 1 conical sensillum.

##### Description.

**Female.** With 13 pairs of legs. Measurements: Body length 2.08 mm, caudal bundle 0.25 mm.

**Figure 4.**
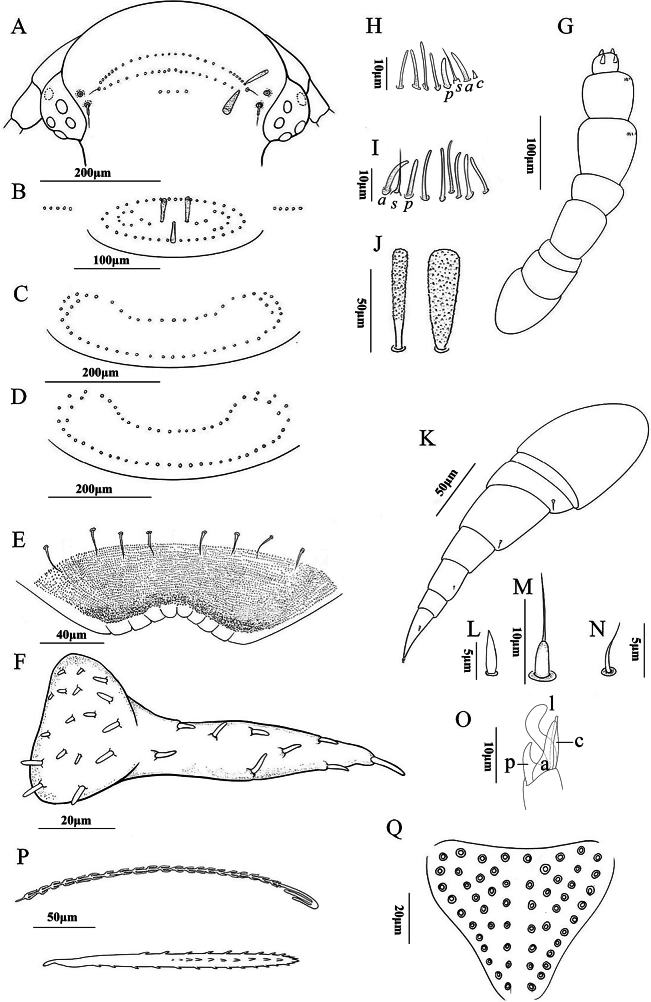
*Polyxenushangzhoensis* Ishii & Liang, 1990 adult female **A** head **B** collum **C** and **D** tergites showcasing the pattern of trichome insertions **C** tergite II **D** tergite III **E** clypeo-labrum **F** gnathochilarium **G** antenna **H** sensilla on articles VII **I** sensilla on articles VI **J** anterior vertex trichome **K** left 13^th^ leg **L** spine on tarsus II **M** typical setae of prefemur and femur **N** seta on tibia **O** telotarsus structure with processes indicated: a: anterior process, c: claw, l: lamella, p: posterior process **P** hooked caudal trichome **Q** pattern of insertions of dorso-medial trichomes on telson. Scale bars: 200 μm (**A, C, D**); 100 μm (**B, G**); 50 μm (**J, K, P**); 40 μm (**E**); 20 μm (**F, Q**); 10 μm (**H, I, M, O**); 5 μm (**L, N**).

***Head*** (Fig. 4A): Eye consisting of 5 ommatidia. Trichomes on the posterior vertex are arranged in 2 rows with no medial gap: the anterior row consists of 26 trichomes, and the posterior row comprises 20 trichomes. Additionally, 4 trichomes are arranged transversely in a dorso-medial position (Fig. 4A). The head features two types of trichomes (Fig. 4J). Three trichobothria are positioned in an equilateral triangle, with the trichobothrium furthest from the ommatidia being slightly smaller than the other two (Fig. 4A). The gnathochilarium possesses lateral palps that are 2.2 times the length of the medial palp. Lateral palps with 9 sensilla, medial palp with 17 sensilla (Fig. 4F). The anterior margin of the labrum is granulated and armed with 5+5 lamellar teeth. The clypeo-labrum is equipped with 8 setae (Fig. 4E).

***Antennae***: Long antennae with proportions of antennal articles as depicted in Fig. 4G. Antennal article VIII with 4 sensory cones, antennal article VI with 2 thick basiconic sensilla (anterior, *a*, and posterior, *p*); 1 setiform sensillum (*s*) between *a* and *p*, 6 thin basiconic sensilla behind *p* (Fig. 4I). Antennal article VII with 2 thick basiconic sensilla (*a* and *p*), 1 setiform sensillum (*s*) between *a* and *p*, 4 thin basiconic sensilla in front of *p*, and 1 conical sensillum (*c*) behind *a* (Fig. 4H).

***Trunk***: Tergal trichomes, including those on the collum, are arranged in a circular pattern. Collum with 54 trichomes, lateral protuberance of collum with 5 trichomes in a row (Fig. 4B). Tergite II with 56 trichomes (Fig. 4C). Tergite III with 60 trichomes (Fig. 4D). Tergites II–X have the same pattern of trichome insertions.

***Legs*** (Fig. 4K): Coxa I with no seta, coxa II with 2 setae, coxae III–XIII with no seta, prefemur and femur with one seta (Fig. 4M), trochanter, post-femur, and tarsus I with no seta. The spine on tarsus II is far shorter than the setae on the prefemur and femur (Fig. 4L). There is a very small seta on the tibia (Fig. 4N). The telotarsus consists of an anterior process with an enlarged base, nearly equaling the length of the claw. Both a lamella process and a small posterior process are present (Fig. 4O).

***Telson***: The telson possesses 54 (27+27) dorso-medial trichomes in the caudal penicil (Fig. 4Q), arranged in two bundles separated by a gap. The caudal bundles consist of two types of trichomes, with the hooked trichomes having an apical lobed hook typical of *Polyxenus* (Fig. 4P).

**Male.** With 13 pairs of legs. Measurements: body length 1.9 mm, caudal bundle 0.22 mm. Posterior vertex with 48 trichomes, Collum with 55 trichomes, and tergites II and III with 62 and 64 trichomes. Coxa I with 1 seta, coxa II with 2 setae, coxae III–XIII with no seta. The caudal penicil comprises 48 (24+24) dorso-medial trichomes.

##### Distribution.

China (Zhejiang, Anhui).

##### Remarks.

This species closely resembles *Polyxenusshinoharai* Ishii, 1983 but differs in the number of trichomes present: the posterior vertex has 47 or 48 (40 or 41 in *P.shinoharai*), the collum with 54 or 55 (41 or 42), the lateral protuberance of the collum bears 4 or 5 (3), tergite II exhibits 56–62 (41 or 43), and tergite III displays 60–64 (42 or 46), antennal article VI with 6 thin basiconic sensilla (7 in *P.shinoharai*).

### ﻿Family Lophoproctidae Silvestri, 1897

#### ﻿Genus Lophoturus

Taxon classificationAnimaliaPolyxenidaPolyxenidae

Brolemann, 1931

048E04C1-93B3-57A2-A3F5-1D94CFF4DD5A

##### Type species.

*Lophoturusobscurus* Brolemann, 1931.

##### Genus diagnosis.

Ommatidia absent. Antennal segment VIII is equal to segment VII. It is characterized by 0 to 4 pairs of linguiform processes on each side of median cleft of labrum and antennal article VI with 3 thick sensilla (Ishii et al. 1999).

##### Included species.

*Lophoturusadisi* Ishii, Nguyen Duy-Jacquemin & Condé, 1999, *L.aequatus* (Loomis, 1936), *L.anisorhabdus* (Condé & Terver, 1964), *L.boondallus* Huynh & Veenstra, 2018, *L.crassipes* Condé & Terver, 1979, *L.danhomenou* (Brolemann, 1926), *L.difficilis* (Condé & Jacquemin, 1963), *L.drifti* (Condé & Terver, 1964), *L.fluctuans* (Condé & Terver, 1964), *L.guineensis* (Silvestri, 1948), *L.hesperius* (Condé & Terver, 1963), *L.humphreysi* Nguyen Duy-Jacquemin, 2014, *L.jianshuiensis* Ishii & Yin, 2000, *L.judsoni* Nguyen Duy-Jacquemin, 2002, *L.longisetis* (Pocock, 1894), *L.scopiger* Condé & Terver, 1979, *L.madecassus* (Marquet & Condé, 1950), *L.monserratensis* Nguyen Duy-Jacquemin, 2002, *L.molloyensis* Huynh & Veenstra, 2018, *L.niveus* (Loomis, 1934), *L.obscurus* (Brolemann, 1931), *L.catalai* (Condé & Nguyen Duy-Jacquemin, 1977), *L. O. kurtchevae* Nguyen Duy-Jacquemin & Condé, 1982, *L.tongae* (Nguyen Duy-Jacquemin & Condé, 1982), *L.okinawai* (Nguyen Duy-Jacquemin & Condé, 1982), *L.peruanus* (Silvestri, 1949), *L.quebradanus* (Chamberlin, 1955), *L.porchi* Huynh & Veenstra, 2020, *L.queenslandicus* (Verhoeff, 1924), *L.speophilus* Nguyen Duy-Jacquemin, 2014, *L.sturmi* Nguyen Duy-Jacquemin, 2002, *L.sineprocessus* sp. nov., *L.vicarius* Condé & Terver, 1979.

#### ﻿ Lophoturus sineprocessus sp. nov.

Taxon classificationAnimaliaPolyxenidaPolyxenidae

46A86B97-F431-53A8-9AA3-AD20F5421397

https://zoobank.org/CDFBBBC5-8162-4CBA-BC54-DD1EA23B3E65

Fig. 5

##### Type material.

***Holotype***: China • ♀; Yunnan, Xishuangbanna Dai Autonomous Prefecture, Jinghong, Menglai Rainforest Health Theme Park; 21°96'63"N, 100°80'55"E; 20 August 2023; Y. D. Wang leg.; GenBank: PQ142933. CBF CZYNS1. ***Paratype***: • 1♂, same data as the holotype.

##### Diagnosis.

Number of trichomes: posterior vertex: 28–36, collum: 76–84, lateral protuberance of collum: 6, tergite II: 82–88, tergite III: 80–84. Antennal article VI with 3 thick basiconic sensilla and 1 conical sensillum; article VII with 2 thick basiconic sensilla. Dorso-medial trichomes on each side consist of 6 sockets of trichome *a*, a single trichome *b*, and two large protruding base sockets of trichome *c*: *c*^1^ and *c*^3^. No linguiform processes on the labrum.

##### Description.

**Female.** With 13 pairs of legs. Measurements: Body length 2.0 mm, caudal bundle 0.38 mm.

**Figure 5.**
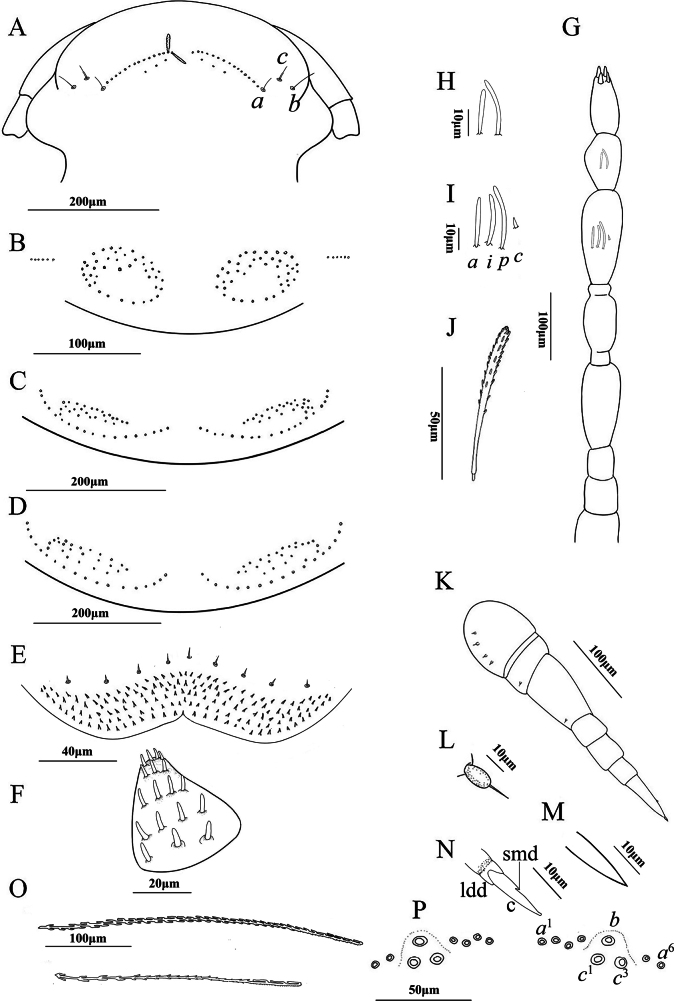
*Lophoturussineprocessus* sp. nov. adult female **A** head **B** collum **C** and **D** tergites showcasing the pattern of trichome insertions **C** tergite II **D** tergite III **E** clypeo-labrum **F** gnathochilarium **G** antenna **H** sensilla on articles VII **I** sensilla on articles VI **J** anterior vertex trichome **K** left 13^th^ leg **L** typical setae of coxa, prefemur, and femur **M** spine on tarsus II **N** telotarsus structure with processes indicated: ldd: latero-dorsal denticles, c: claw, smd: small denticle **O** hooked caudal trichome **P** pattern of insertions of dorso-medial trichomes on telson. Scale bars: 200 μm (**A, C, D**); 100 μm (**B, G, K, O**); 50 μm (**J, P**); 40 μm (**E**); 20 μm (**F**); 10 μm (**H, I, L, M, N**).

***Head*** (Fig. 5A): Ommatidia absent. The posterior vertex has one pair of tufts arranged in two rows, with the anterior row consisting of 14 trichomes and the posterior row of 4 (Fig. 5A). Trichomes are depicted in Fig. 5J. Three trichobothria are arranged in an isosceles triangle, trichobothria *a* and *b* have typically thin sensory hairs with narrow cylindrical funicles compared to trichobothrium *c*, with a claviform funicle (Fig. 5A). The gnathochilarium is typical of Lophoproctidae, featuring a single medial palp with 18 sensilla (Fig. 5F). The clypeo-labrum possesses 4+1+4 setae and lacks linguiform processes on each side of the median cleft of the labrum (Fig. 5E).

***Antennae***: Long antennae with proportions of antennal articles as depicted in Fig. 5G. Antennal article VIII with 4 sensory cones; antennal article VI with 3 thick basiconic sensilla (*a*, *i*, and *p*) and 1 conical sensillum (*c*) (Fig. 5I); article VII with 2 thick basiconic sensilla (Fig. 5H).

***Trunk***: Collum, each with one pair of tufts consisting of 42 trichomes, lateral protuberance of collum with 6 trichomes in a row (Fig. 5B). Tergite II, each with one pair of tufts consisting of 44 trichomes (Fig. 5C). Tergite III, each with one pair of tufts consisting of 42 trichomes (Fig. 5D). Tergites II–X exhibit consistent patterns of trichome insertions.

***Legs*** (Fig. 5K): Trochanter, post-femur, tibia, and tarsus I lack setae. Prefemur and femur each with 1 seta, coxa I with 1–2 setae, coxae II–XIII with 3–4 setae (Fig. 5L), spine on tarsus II slightly shorter than telotarsus (Fig. 5M). The telotarsus with two latero-dorsal denticles, a claw, and a small denticle (Fig. 5N).

***Telson***: Dorso-medial trichomes on each side with 6 sockets of trichome *a*, a single trichome *b*, and two large protruding base sockets of trichome *c*: *c*^1^ and *c*^3^ (Fig. 5P, the absence of *c*^2^ is characteristic of Lophoproctidae species). Two bundles of caudal trichomes are unseparated. The telson trichomes are of two types, both exhibiting barbs (Fig. 5O).

**Male.** With 13 pairs of legs. Measurements: Body length 1.8 mm, caudal bundle 0.3 mm. The posterior vertex possesses one pair of tufts arranged in two rows, with the anterior row consisting of 12 trichomes and the posterior row containing 2 trichomes. The gnathochilarium features 32 sensilla. The collum exhibits one pair of tufts, each consisting of 38 trichomes. Tergites II and III each bear one pair of tufts comprising 41 or 40 trichomes, respectively. Coxa I with 2 setae, coxa II with 3 setae, coxae III–VII with 4 setae, coxae VIII–XII with 2–3 setae, coxa XIII with no seta.

##### Distribution.

China (Yunnan).

##### Etymology.

The species name is derived from the absence of linguiform processes on each side of the median cleft of the labrum, a distinctive characteristic of the species.

##### Remarks.

The new species resembles *Lophoturusjianshuiensis* Ishii & Yin, 2000 but differs in the following aspects: absence of linguiform processes on each side of the median cleft of the labrum (*L.jianshuiensis* has 1 pair of linguiform processes), female gnathochilarium with 18 sensilla (30 or 31 sensilla), dorso-medial trichomes on each side with 6 sockets of trichome *a* (5 sockets of trichome *a*).

### ﻿Phylogenetic results

**Figure 6.**
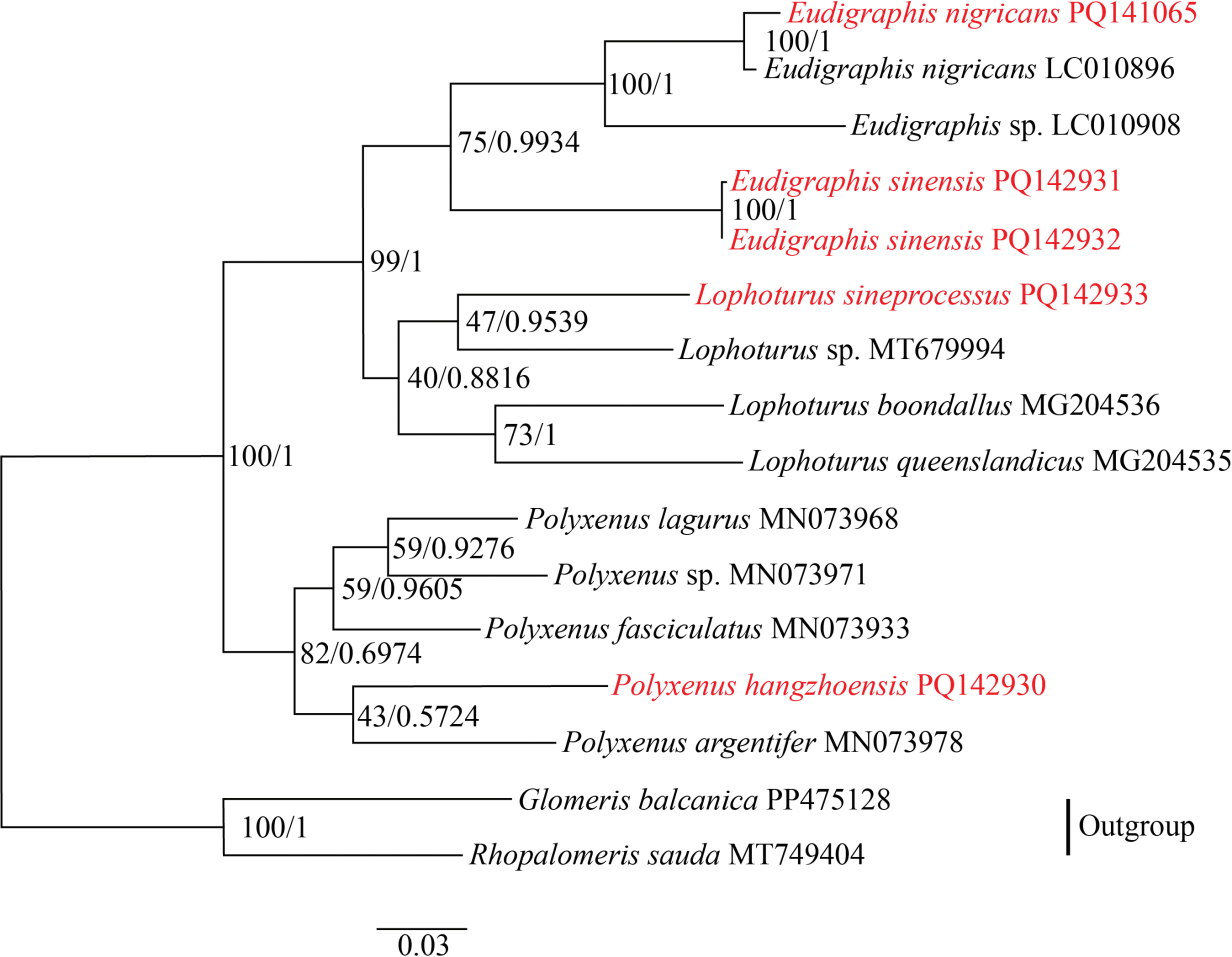
Unrooted phylogenetic tree based on cytochrome *c* oxidase subunit I (COI) sequence data. Bootstrap proportions of ML and Bayesian posterior probability (BPP) are shown at each node (BP/BPP). The species sequenced in this study are indicated in red.

ML and BI trees have been constructed (Fig. 6). Most nodes exhibit strong support. The ML and BI tree topologies were identical, supporting *Polyxenus*, *Lophoturus*, and *Eudigraphis* as monophyletic groups. The newly sequenced species: *Eudigraphisnigricans* (Miyosi, 1947), *Eudigraphissinensis* Ishii & Liang, 1990, *Polyxenushangzhoensis* Ishii & Liang, 1990, and *Lophoturussineprocessus* sp. nov., clustered within their respective genera.

## ﻿Discussion

Since Linnaeus first recorded Polyxenida in 1758 (Linnaeus 1758), this group has garnered significant attention from taxonomists worldwide due to their unique morphological characteristics and systematic position. However, research has been more substantial in Europe, the Americas, and Australia compared to Asia and Africa. Currently, there are 32 described species in the genus *Lophoturus* worldwide (Huynh and Veenstra 2018b; Huynh and Veenstra 2020), with China having two species. The genus *Monographis* comprises 16 described species worldwide (Huynh and Veenstra 2013; Huynh and Veenstra 2022; Huynh et al. 2023), with China having two species. The genus *Eudigraphis* includes six described species worldwide (Karasawa et al. 2020), with China having three species. The genus *Polyxenus* consists of 30 described species worldwide (Enghoff et al. 2015; Short et al. 2020), with China having three species. The understanding of Polyxenida species diversity in China remains limited, and there is a pressing need for in-depth and systematic investigations.

This study collected four Polyxenida species from four regions in China. Comprehensive morphological descriptions and molecular phylogenetic tree construction corroborated the inclusion of one new species and one newly recorded species, increasing the known Polyxenida species in China from 10 to 12. However, the increasing scarcity of classical taxonomists, coupled with factors such as phenotypic plasticity and genetic variation, presents objective difficulties and confusion in the accurate identification of biological samples. Molecular markers, such as cytochrome COI, have been widely used in other groups to assist in species identification. Therefore, it is imperative to integrate classical taxonomy with molecular identification techniques and to standardize their application in the classification of Polyxenida and other soil fauna.

### ﻿Key to the species of Penicillata from China

**Table:** 

1	Antennal segment VIII equal to segment VII (Fig. 5G)	**2**
–	Antennal segment VIII shorter than segment VII (Figs 2G, 3G, 4G)	**3**
2	Two pairs of linguiform processes on each side of the median cleft of labrum	***Lophoturusokinawai* (Nguyen Duy-Jacquemin & Condé, 1982)**
–	One pair of linguiform processes on each side of the median cleft of labrum	***Lophoturusjianshuiensis* Ishii & Yin, 2000**
–	No linguiform processes on each side of the median cleft of labrum (Fig. 5E)	***Lophoturussineprocessus* sp. nov.**
3	Fan of barbate trichomes present dorso-medially, anterior to penicil, with the two bundles of trichomes forming the caudal penicil being widely separated	**4**
–	Dorso-medial fan of barbate trichomes absent, two bundles of caudal penicil closely aligned, giving the appearance of a single bundle	**6**
4	Ommatidia present	**5**
–	Ommatidia absent	***Polyxenusanophthalius* Ishii & Yin, 2000**
5	Three ommatidia	***Polyxenustriocellatus* Ishii & Yin, 2000**
–	Five ommatidia (Fig. 4A)	***Polyxenushangzhoensis* Ishii & Liang, 1990**
6	The spine of the tarsus is thick and conical in shape	**7**
–	Tarsal spine absent, small setiform hair with round base present	**8**
7	Antennal segment VI with short thick basiconic sensilla, anterior margin of labrum with a line of marginal setae	***Monographisbaihualingensis* Ishii & Yin, 2000**
–	Antennal segment VI without short thick basiconic sensilla, anterior margin of labrum without a line of marginal setae	***Monographisyunnanensis* Ishii & Yin, 2000**
8	Labrum with 3 + 3 lamellar teeth (Fig. 3E)	**9**
–	Labrum with 4 + 4 lamellar teeth	***Eudigraphisxishuangbanna* Ishii & Yin, 2000**
–	Labrum with 2 + 2 lamellar teeth	***Eudigraphistaiwaniensis* Ishii, 1990**
9	Head black	***Eudigraphisnigricans* (Miyosi, 1947)**
–	Head pale brown with cream yellow	***Eudigraphissinensis* Ishii & Liang, 1990**

## Supplementary Material

XML Treatment for Eudigraphis

XML Treatment for Eudigraphis nigricans

XML Treatment for Eudigraphis sinensis

XML Treatment for Polyxenus

XML Treatment for Polyxenus hangzhoensis

XML Treatment for Lophoturus

XML Treatment for Lophoturus sineprocessus

## ﻿Additional information

### Author ORCIDs

Yadong Wang https://orcid.org/0009-0003-6209-5746

Ai Jin https://orcid.org/0009-0009-9221-3014

Shichen Gao https://orcid.org/0000-0002-4628-960X

Jiajia Wang https://orcid.org/0000-0002-1843-3977

Yan Dong https://orcid.org/0000-0001-6562-2511

### Data availability

All data supporting the findings of this study are available in the main text or the National Center for Biotechnology Information (NCBI) GenBank database (https://www.ncbi.nlm.nih.gov/Genbank) under accession numbers PQ141065, PQ142930, PQ142931, PQ142932, and PQ142933.
